# Longitudinal change of gut microbiota in hypertensive disorders in pregnancy: a nested case–control and Mendelian randomization study

**DOI:** 10.1038/s41598-023-43780-w

**Published:** 2023-10-09

**Authors:** Xinrui Wu, Qi Li, Jiawang Cai, Houxiang Huang, Shujuan Ma, Hongzhuan Tan

**Affiliations:** 1 School of Medicine, Jishou University, Jishou, China; 2 Xiangya School of Public Health, Central South University, Changsha, China; 3 Xiangxi Center for Disease Control and Prevention, Jishou, China; 4 Reproductive and Genetic Hospital of CITIC-Xiangya, Changsha, China

**Keywords:** Hypertension, Pre-eclampsia, Bacteria

## Abstract

Mounting evidence has shown that gut microbiota (GM) is related to hypertensive disorders in pregnancy (HDP), however, most studies only focused on one time point in pregnancy. In this study, we conducted a nested case–control study utilizing a follow-up cohort, resulting in the collection of 47 HDP patients and 30 healthy controls. The GM profiles were explored using 16S rRNA sequencing at three time points during pregnancy. The diversity analysis of GM showed no significant difference between HDP patients and controls, however, we found 21 differential GM during pregnancy. Trend analysis showed that there are statistical differences in the relative abundance of *Thermomonas*, *Xanthomonas*, and *Phenylobacteriumat* during pregnancy in the gestational hypertension group, and of *Xanthomonas*, *Polycyclovorans*, and *Phenylobacterium* in the control group. The correlation study found that six genera of GM are related to blood pressure. Furthermore, the MR analysis identified the causal relationship between *Methanobrevibacter* and pre-eclampsia (PE). This study first explored the longitudinal change of GM in HDP patients during pregnancy, found the differential GM, and detected the causal association. Our findings may promote the prevention and treatment of HDP from the perspective of GM and provide valuable insights into the pathogenesis of HDP.

## Introduction

Hypertensive disorders in pregnancy (HDP), which mainly include gestational hypertension (GH) and pre-eclampsia (PE), are serious complications in pregnant women. Specifically, GH is characterized by the first elevation in blood pressure (systolic ≥ 140 and/or diastolic ≥ 90 mm Hg) after 20 weeks of gestation, while PE is diagnosed when a woman with GH also has increased protein in her urine^1^. At present, the incidence rate of HDP is about 7% with up to 60,000 fatalities resulting from HDP annually^2^, making it the second leading cause of maternal mortality^3^. Previous studies found that HDP can lead to enduring medical impairments for pregnant women, such as hypertension, diabetes, cardiovascular disease, and ischemic stroke^4–6^. In addition, the risk of hypertension, abnormal blood lipid metabolism, cognitive impairment, and mental disorders in the offspring of HDP patients would also significantly increase^7–9^. Despite several years of investigation, the pathogenesis of HDP remains incompletely elucidated. Therefore, exploring the mechanism of HDP from a novel perspective is imperative for its prevention and treatment.

Gut microbiota (GM) changes significantly during gestation, and the dysbiosis in GM may contribute to physiological dysfunction in pregnancy^10,11^. Increasing evidence has demonstrated the association between GM and HDP^12,13^. For example, Altemani et al. found that the decreased abundance of *Coprococcus* may elevate the risk of PE in pregnant women^12^. Another case–control study demonstrated a reduction in bacterial diversity and beneficial genera, such as *Faecalibacterium* and *Akkermansia* in patients with PE^13^. However, these findings had some limitations. Firstly, the human intestinal environment is complex and frequently influenced by multiple factors, making it difficult to regulate potential confounding factors. Secondly, most existing results came from observational studies, and the timing of exposure and outcome remains unclear to make the causal inference. Furthermore, previous studies are mainly focused on one time point in pregnancy^14,15^, ignoring to explore the composition and longitudinal change of GM during the early, middle, and late trimesters.

Mendelian randomization (MR) is a useful approach using genetic variants as instrumental variables (IVs) to make causal inferences^16^. Because the alleles from parents to offspring are randomly assigned, freely combined and the genotypes remain stable after birth, it can reduce confounding factors as well as exclude reverse causality, which is regarded as the “most natural” randomized controlled trial (RCT)^17,18^. Many studies have used MR analysis to explore the correlation between GM and some complex human diseases including metabolic diseases, anxiety disorders, and neurogenerative diseases^19–21^.

At present, the mechanism of HDP has not been fully elucidated, and the current researches are limited to cross-sectional design, lacking prospective study. Given the evidence of dynamic alterations in GM during pregnancy and the strong association between GM and HDP, we hypothesized that the change of GM during pregnancy may participate in the occurrence and development of HDP. In this study, we performed a prospective nested case–control study to characterize and analyze the differences in the composition and longitudinal alternation of GM between HDP (GH/PE) patients and controls during the early, middle, and late trimesters. Then, using MR analysis to further evaluate the causal association between differential GM taxa and HDP. Our findings may promote the prevention and treatment of HDP from the perspective of GM and provide new insights into the pathogenesis of HDP.

## Results

### Characteristics of the subjects

A total of 46 HDP patients (29 GH cases and 17 PE cases) and 30 controls with normal blood pressure during pregnancy were included in this study. On average, GH cases and PE cases were diagnosed at 36.21 and 32.75 weeks of gestation of pregnancy, respectively. The baseline data revealed that the average age of HDP patients was 31.11 (± 4.01) years old, while that of the control group was 31.30 (± 3.47) years old with no significant difference, but the admission age of PE was higher than that of GH (*P* < 0.01). The pre-pregnancy BMI in the HDP group was significantly higher than that in the control group (*P* = 0.002). There was also a significant difference in pre-pregnancy BMI among GH, PE, and control groups (*P* < 0.001). Other characteristics of subjects including primipara, smoking history, alcohol drinking history, and blood pressure in each trimester among different groups were demonstrated in Table 1.

**Table 1:** Characteristics of the subjects.

	Controls	Cases		
		HDP	GH	PE
Baseline				
Sample size	30	46	29	17
Age	31.30 (± 3.47)	31.11 (± 4.01)	29.59 (± 3.30)	33.71 (± 3.85)^#^
BMI before pregnancy	20.54 (± 2.03)	22.51 (± 3.30)*	21.46 (± 2.83)	24.30 (± 3.35)*^#^
Primipara				
Yes	11 (36.7%)	24 (52.2%)	16 (55.2%)	8 (47.1%)
No	19 (63.3%)	22 (47.8%)	13 (44.8%)	9 (52.9%)
Smoking history				
Yes	1 (3.3%)	1 (2.2%)	1 (3.4%)	0
No	29 (96.7%)	45 (97.8%)	28 (96.6%)	17 (100%)
Alcohol drinking history				
Yes	2 (6.7%)	1 (2.2%)	0	1 (5.9%)
No	28 (93.3%)	45 (97.8%)	29(100%)	16 (94.1%)
Early pregnancy				
Sample size	30	43	27	16
Gestational age of stool sample(weeks)	13.44 (± 0.93)	13.54 (± 1.13)	13.62 (± 0.97)	13.42 (± 1.39)
SBP (mm Hg)	113.80 (± 9.58)	123.91 (± 8.05)*	123.07 (± 8.02)*	125.31 (± 8.15)*
DBP (mm Hg)	71.63 (± 6.67)	81.05 (± 6.51)*	80.11 (± 6.11)*	82.63 (± 7.05)*
Middle pregnancy				
Sample size	30	35	24	11
Gestational age of stool sample(weeks)	24.79 (± 0.98)	25.12 (± 1.23)	25.32 (± 1.27)	24.68 (± 1.06)
SBP (mm Hg)	108.63 (± 8.13)	147.06 (± 12.76)*	146.88 (± 13.35)*	147.46 (± 7.88)*
DBP (mm Hg)	69.17 (± 5.57)	91.09 (± 5.76)*	90.42 (± 5.86)*	92.59 (± 5.63)*
Late pregnancy				
Sample size	30	29	22	7
Gestational age of stool sample(weeks)	37.21 (± 0.74)	37.36 (± 1.14)	37.47(± 1.00)	37.02 (± 1.54)
SBP (mm Hg)	106.70 (± 7.06)	144.59 (± 11.23)*	144.36 (± 12.53)*	145.29 (± 10.38)*
DBP (mm Hg)	69.20 (± 6.56)	92.37 (± 8.97)*	91.50 (± 8.06)*	95.29 (± 9.67)*

*Compared with the control group, the difference was statistically significant (*P* < 0.05).

^#^There was significant difference between the two case groups (GH and PE) (*P* < 0.05).

*HDP* hypertensive disorders in pregnancy, *GH* gestational hypertension, *PE* pre-eclampsia, *BMI* body mass index, *SBP* systolic pressure, *DBP* diastolic pressure.

### 16S rRNA sequencing analysis

In this study, a total of 16,257,425 original Tags data with an average of 82,525 reads per sample were obtained after sequencing. Quality control was carried out based on the QIIME2, and 15,602,597 high-quality reads with an average length of 253 bp per sample were collected for subsequent analyses. Rarefaction curves showed that the amount of sequencing can fully reflect the microbial diversity of the samples (Figure S1).

### Composition and diversity analysis

**Figure 1:**
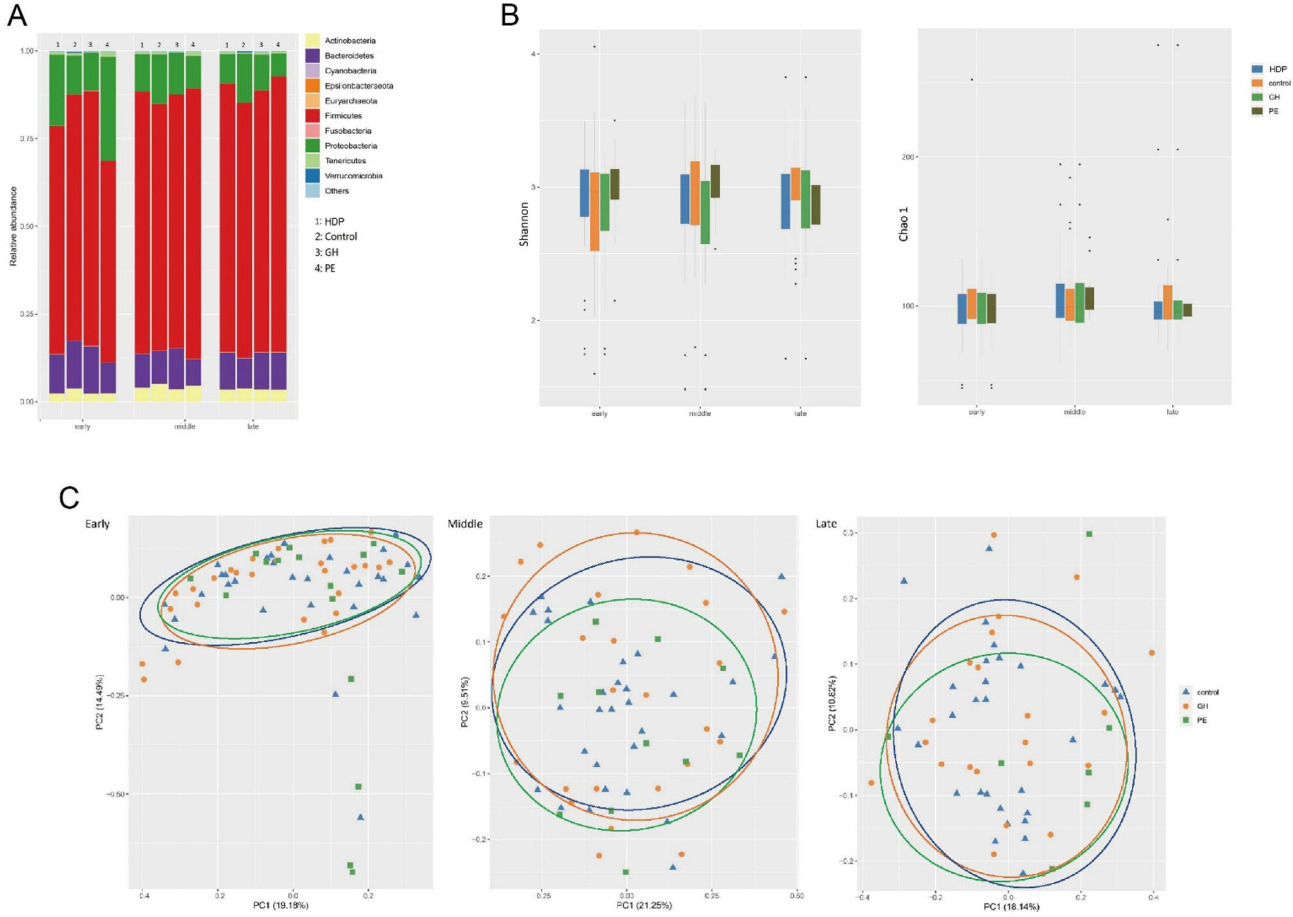
Composition and diversity analysis of GM in three trimesters of pregnancy. (**A**) Composition analysis; (**B**) alpha diversity; (**C**) the principal coordinates analysis (PCoA) on beta diversity. GM, gut microbiota.

At the phyla level, the five most abundant taxa at the phylum level were *Firmicutes*, *Proteobacteria*, *Bacteroidetes*, *Actinobacteria*, and *Tenericutes*, with the top three accounting for approximately 95% of the total gut microbiota. Figure 1A showed the distribution of the top 10 microbiota of GH, PE, and the control group during the early, middle, and late trimesters.

The Shannon index for the control, GH, and PE groups during the three trimesters were similar with the average ranging from 2.81 to 3.01. While the Chao1 index exhibited slight fluctuations, there were no significant differences observed among the groups and trimesters in terms of these two alpha diversity indices (Table S1, Fig. 1B). In addition, the PERMANOVA analysis revealed no significant differences in beta diversity among GH, PE, and control groups during the early, middle, and late trimester (Fig. 1C).

### Microbial taxa alternation

**Figure 2:**
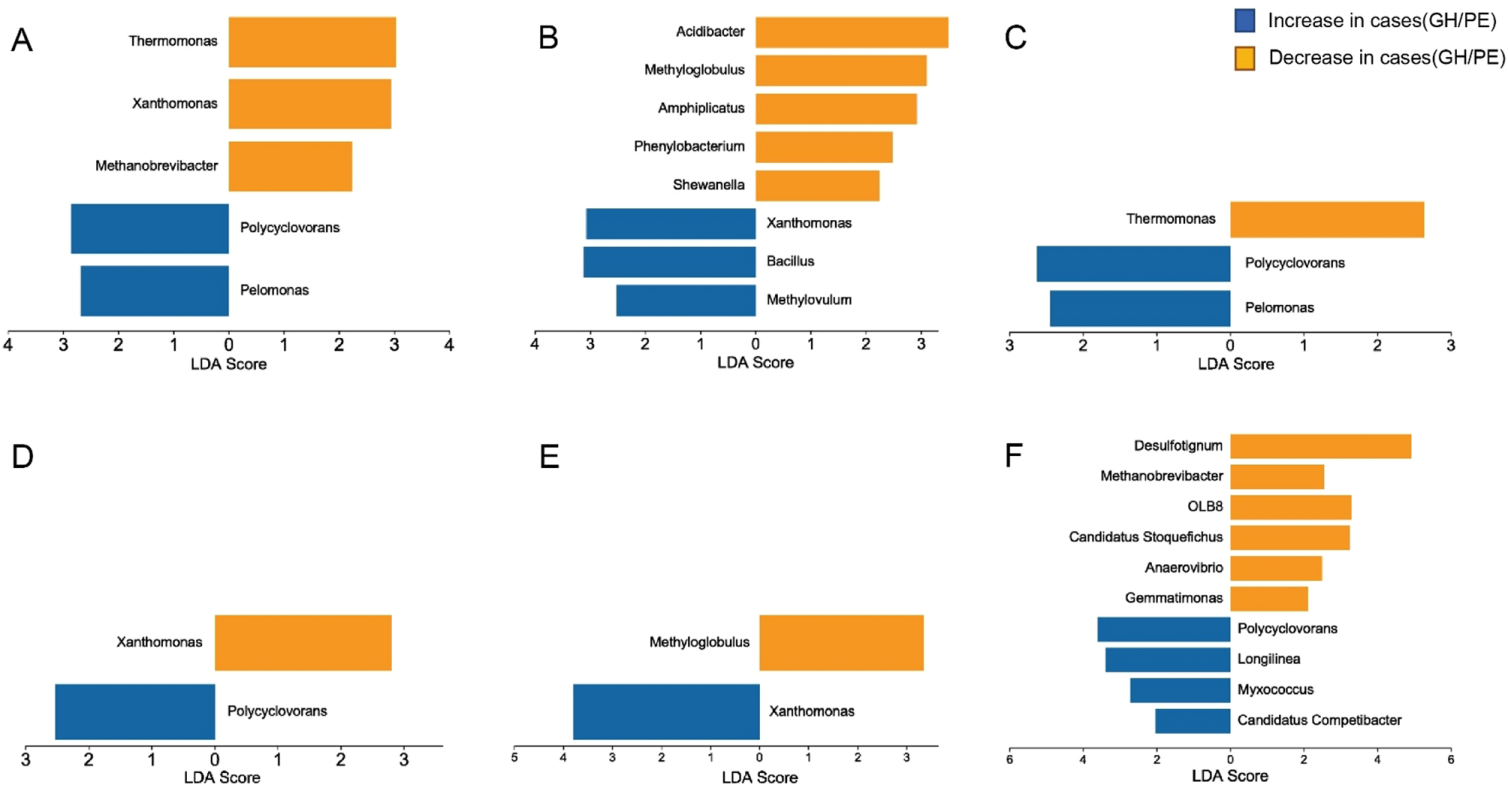
Linear discriminant analysis (LDA) score of differential taxa at genus level based on LEfSe (*P* < 0.05 and LDA threshold value > 2) in three trimesters of pregnancy. (**A**) GH cases/controls in the early trimester; (**B**) GH cases/controls in the middle trimester; (**C**) GH cases/controls in the late trimester; (**D**) PE cases/controls in the early trimester; (**E**) PE cases/controls in the middle trimester; (**F**) PE cases/controls in the late trimester. GH, gestational hypertension; PE, pre-eclampsia.

The LEfSe analysis was performed to explore the differential GM in HDP patients (GH and PE) compared with the healthy pregnant women in different pregnancy periods (Table S2, Fig. 2). In the early trimester, *Polycyclovorans* (*P* < 0.001) and *Pelomonas* (*P* = 0.032) were significantly more abundant in the women who were later diagnosed with GH, whereas *Thermomonas* (*P* = 0.002), *Xanthomonas* (*P* < 0.001), and *Methanobrevibacter* (*P* = 0.047) were considerably enriched in the healthy controls. Women who were later diagnosed with PE are more enriched in *Polycyclovorans* (*P* = 0.001) and have a lower abundance of *Xanthomonas* (*P* = 0.001). In the middle trimester, women who were later diagnosed with GH or PE both showed a significantly elevated abundance of *Xanthomonas* and a lower abundance of *Methyloglobulus* than healthy controls. Additionally, the GH group was significantly enriched for *Bacillus* (*P* = 0.047) and *Methylovulum* (*P* = 0.005). In the late trimester, women who were later diagnosed with GH were significantly enriched for *Polycyclovorans* (*P* = 0.021) and *Pelomonas* (*P* = 0.031), while the healthy controls showed a significantly increased abundance of *Thermomonas* (*P* = 0.007). Women who were later diagnosed with PE had a higher abundance of *Polycyclovorans* (*P* = 0.005), *Longilinea* (*P* = 0.019), *Myxococcus* (*P* = 0.022), and *Candidatus Competibacter* (*P* = 0.002) as well as a lower abundance of *Desulfotignum* (*P* = 0.029), *Methanobrevibacter* (*P* = 0.003), *OLB8* (*P* = 0.001), *Candidatus Stoquefichus* (*P* = 0.040), *Anaerovibrio* (*P* = 0.019), and *Gemmatimonas* (*P* = 0.026).

### Trend and correlation analysis in differential GM

There were 19 cases of GH and 3 cases of PE with complete stool samples (during the early, middle, and late trimesters of pregnancy). Due to the limited number of complete samples in the PE group, only the abundance trends of 12 differential bacteria between GH and the control were analyzed during gestation. The results of trend analyses showed that there were significant differences in the relative abundance of *Thermomonas* (*P* = 0.007), *Xanthomonas* (*P* < 0.001), and *Phenylobacterium* (*P* = 0.013) at three time-points of pregnancy in the GH group, and of *Xanthomonas* (*P* = 0.003), *Polycyclovorans* (*P* = 0.002), and *Phenylobacterium* (*P* = 0.001) in the control group (Table S3).

Spearman rank correlation analysis was carried out between the differential bacteria and the blood pressure to test the possible correlation (Figure S2). We observed that, in the early trimester, GH-enriched genus *Polycyclovorans* (*r* = 0.245, *P* = 0.037) was positively correlated with diastolic pressure (DBP). In the middle trimester, GH-decreased genera, including *Acidibacter* (*r* = − 0.281, *P* = 0.023), *Amphiplicatus* (*r* = − 0.248, *P* = 0.047), and *Shewanella* (*r* = − 0.296, *P* = 0.017) were negatively correlated with DBP. Furthermore, in the late trimester, GH-enriched genus *Pelomonas* (*r* = 0.368, *P* = 0.004) was positively correlated with systolic pressure, while GH-decreased genus *Thermomonas* (*r* = − 0.279, *P* = 0.034) was negatively correlated with DBP.

### MR analysis

**Figure 3:**
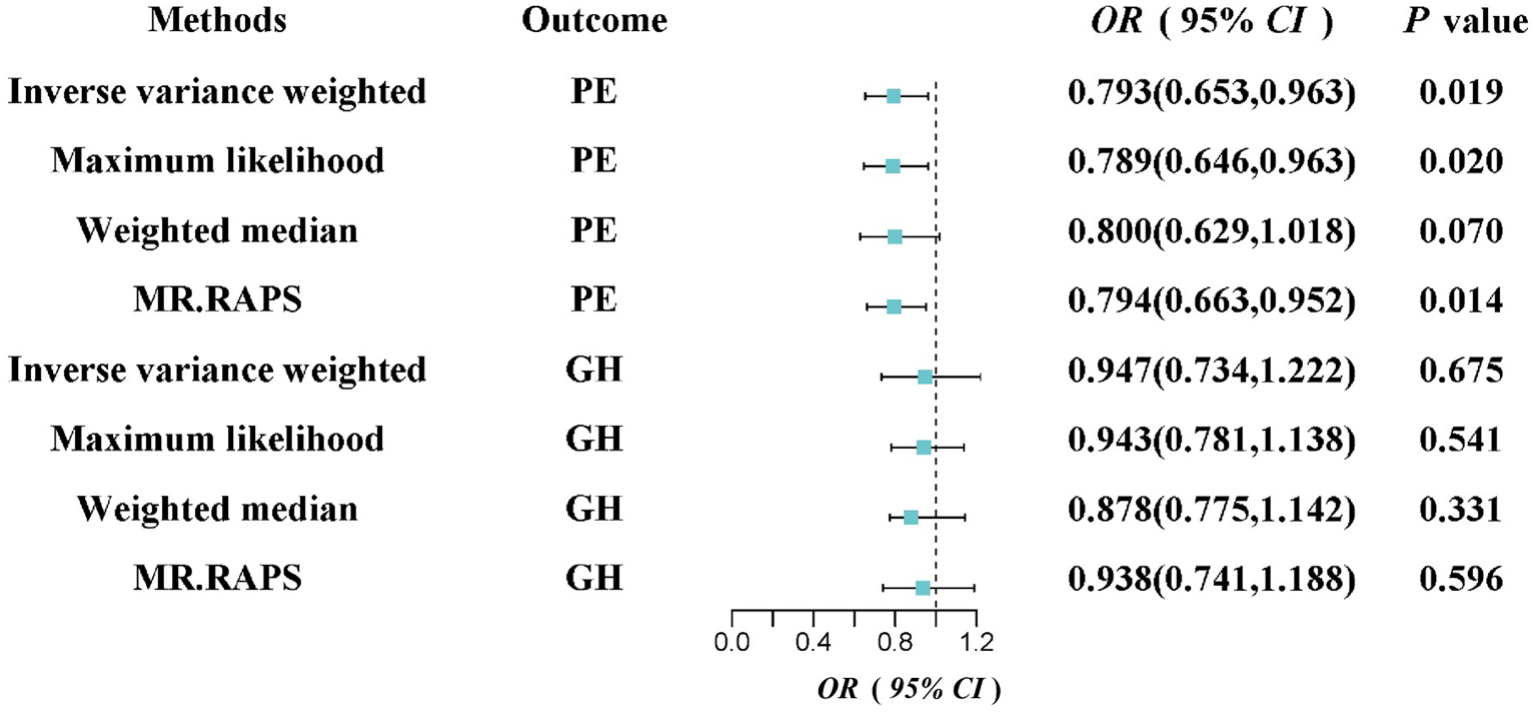
Forrest plot to visualize the causal effect of the GM genus *Methanobrevibacter* on the risk of HDP (PE/GH) by using different MR methods. GM, gut microbiota; HDP, hypertensive disorders in pregnancy; PE, pre-eclampsia; GH, gestational hypertension.

In this study, we identified 21 distinct microbial taxa that exhibited differential abundance in HDP patients (GH and PE) across different pregnancy periods. However, after applying rigorous IV selection criteria, only the genus *Methanobrevibacter* with 6 SNPs was deemed suitable for MR analysis (Table S4). Using the IVW method, we detected a causal relationship between increased *Methanobrevibacter* and reduced risk of PE (OR = 0.793, 95% CI 0.653–0.963, *P* = 0.019), with an *F*-statistic of 179.04. Additionally, both MaxLik (OR = 0.789, 95% CI 0.646–0.963, *P* = 0.020) and MR.RAPS (OR = 0.794, 95% CI 0.663–0.952, *P* = 0.014) estimates provided further evidence supporting the causal association between *Methanobrevibacter* and PE. While no causal association was found between *Methanobrevibacter* and GH (Figure 3). So, we proceeded to utilize *Methanobrevibacter* as the exposure and PE as the outcome for further analyses.

**Figure 4:**
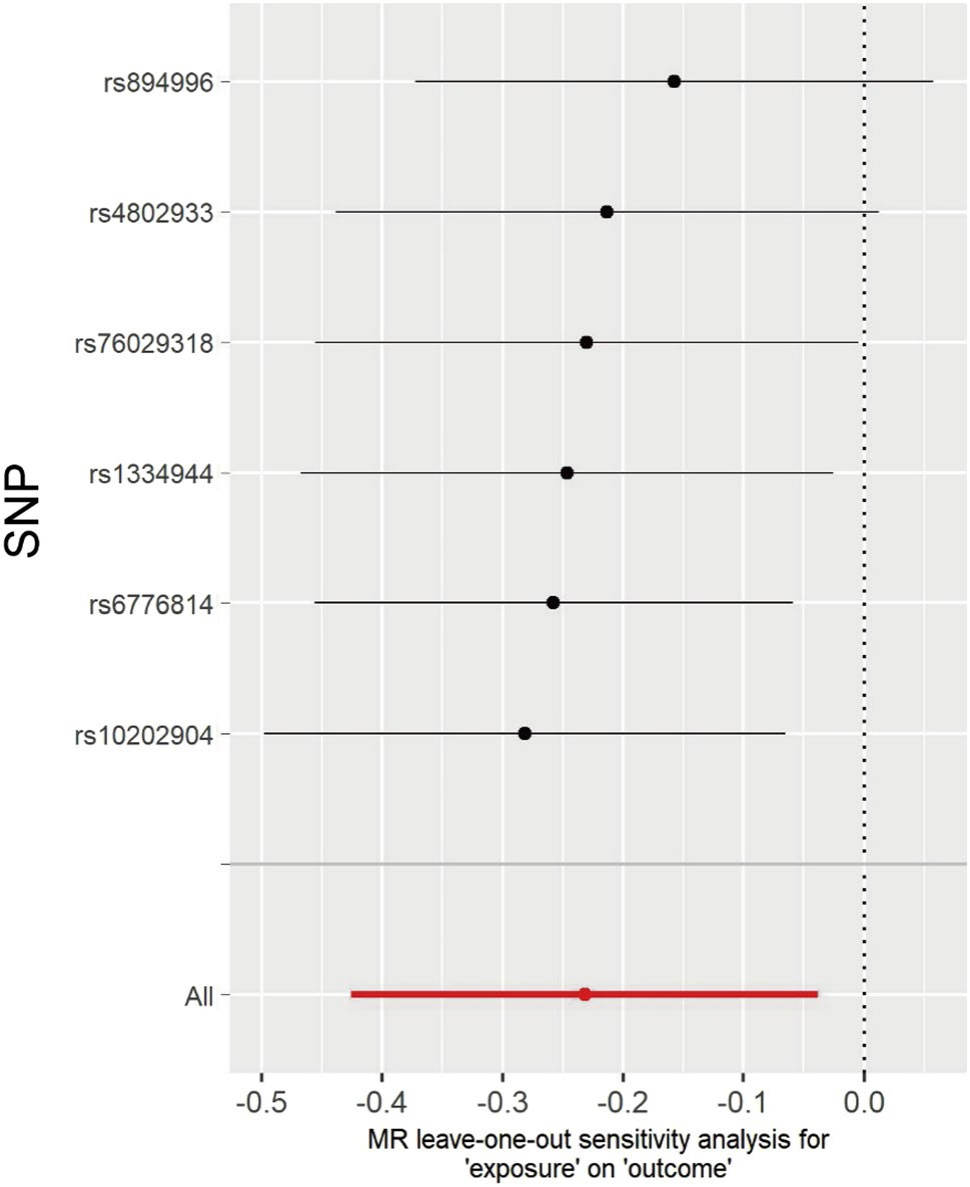
Leave-one-out analysis of the causal effect of the GM genus Methanobrevibacter on the risk of PE. The black points indicate causal effect estimates for the remaining SNPs post the exclusion of this single SNP, while the red point shows the causal effect estimated using all SNPs. Horizontal lines denote the 95% confidence interval. GM, gut microbiota; PE, pre-eclampsia; SNP, single nucleotide polymorphism.

Cochran’s Q statistics showed no significant heterogeneity in selected IVs (*P* = 0.493 in the IVW method and *P* = 0.505 in the MR-Egger method). The MR-Egger intercept (intercept = 0.055, *P* = 0.357) confirmed that there is no significant directional horizontal pleiotropy. Additionally, the forest plot suggested that rs6776814 might be the potential outlier SNP (Figure S3). However, further MR-PRESSO global test (RSSobs = 6.128, *P* = 0.555) and the leave-one-out analysis revealed that there are no outlier IVs that would have a significant impact on the result (Fig. 4).

We conducted the reverse MR analysis to assess whether PE causally affects the genus *Methanobrevibacter*, and the results by using different MR methods confirmed no reverse causation exists (*P* > 0.05, Table S5). Additionally, MVMR results demonstrated that after adjusting for alcohol drinking (OR = 0.770, 95% CI 0.655–0.906, *P* = 0.048), smoking (OR = 0.654, 95% CI 0.497–0.806, *P* = 0.002), and T2D (OR = 0.761, 95% CI 0.642–0.902, *P* = 0.001), *Methanobrevibacter* remained causally associated with PE risk and had a more substantial effect than univariate MR. But after adjusting for BMI (OR = 0.769, 95% CI 0.667–0.880, *P* = 0.067), no causal association existed.

## Discussion

In the nested case–control study, we conducted sequencing of the 16S rRNA gene for gut microbiota DNA from a total of 197 stool samples, comprising 46 HDP patients and 30 healthy controls, and investigated the changes in bacterial composition during early, middle, and late pregnancy. Followed by a bidirectional MR analysis, we further confirmed the causal relationship between the differential microbial genus *Methanobrevibacter* and PE.

Gut microbiota (GM) changes significantly during gestation, previous studies found that the dysbiosis in GM may associated with hormonal change, lifestyle modification, and the increase in maternal body weight^22,23^. In our study, the alpha and beta diversity of GM in HDP (GH and PE) did not exhibit any significant differences, which suggested that the diversity and structure of GM during pregnancy have limited impact on the occurrence and development of HDP. It is not the composition of the whole GM, but maybe some certain taxa that change during pregnancy and have effects on HDP. To investigate this possibility, we conducted the LEfSe analysis to identify the differential microbial taxa in HDP patients across different pregnancy periods. At the genus level, our study found that the relative abundance of *Polycyclovorans* and *Pelomonas* in GH/PE in early pregnancy was significantly higher than that in healthy control, and these bacteria were also enriched in the late trimester of GH/PE patients. *Polycyclovorans* and *Pelomonas* were commonly found in aquatic environments^24,25^, with the latter being identified as enriched in bladder cancer parenchyma^26^, intestinal mucosa of Crohn's disease patients^27^, and non-pregnant women's endometrium^28^. In the middle trimester, differential GM including *Shewanella*, *Bacillus*, *Acidibacter*, *Methyloglobulus*, and so on. Correlation analysis also demonstrated a negative association between *Shewanella* and DBP. *Shewanella*, commonly known as an environmental bacteria, has recently been reported to play an important role in multiple drug resistances^29^. A cohort study conducted on high-risk bladder cancer patient found that *Bacillus* was associated with high blood pressure, this finding was also confirmed by another in vivo study, which both supported our result^30,31^. In the late trimester, the relative abundance of *Anaerovibrio* was significantly decreased in PE patients, which is a short-chain fatty acid (SCFA) butyrate-producing bacteria^32^. SCFAs can provide sufficient nutrients to intestinal cells, assist in the maintenance of normal intestinal permeability, and effectively reduce blood pressure^33,34^. In vivo and in vitro experiments found that butyrate significantly reduces the effects of lipopolysaccharide, thereby promoting macrophage 1 polarization and inhibiting macrophage 2 polarization, ultimately leading to a reduction in blood pressure^35,36^. Furthermore, Jin et al.^37^ reported that butyrate promotes the effect on macrophage autophagy by decreasing autophagy receptors like P62 level and elevating LC3-II/LC3-I ratio, thus alleviating PE symptoms in rats. All the evidence above supported the protective role of *Anaerovibrio* on PE and explained the possible mechanism. There have been relatively few existing researches on *Thermomonas*, *Xanthomonas*, and *Phenylobacterium* which showed significant differences at three time points during pregnancy. So, more functional experiments and RCTs are needed to support their effects on the risk of HDP.

Interestingly, our MR analysis found *Methanobrevibacter* is negatively correlated with PE and this association is still significant after adjusting for covariates such as alcohol drinking, smoking, and T2D. This finding is consistent with two other MR studies^38,39^ and is further supported by our nested case–control study, which demonstrates that the relative abundance of *Methanobrevibacter* is higher in healthy controls than in HDP cases during both early and late pregnancies. Trimethylamine-*N*-oxide (TMAO), a gut microbiota-derived metabolite, has been found to be significantly enriched in PE patients and identified as a contributing factor to cardiovascular diseases in observational studies^40,41^. Previous studies have demonstrated that TMAO has the potential to modify the metabolism of sterols and cholesterol across various bodily locations and processes, leading to an increase in atherosclerosis^42^. Furthermore, individuals with PE have been observed to exhibit “atherosclerosis-like” lesions in the spiral arteries of their placentas, accompanied by lipid accumulation^43^. The rats model also supported the role of TMAO-induced reactive oxygen species in remodeling spiral arterial defects in the placenta, which may contribute to the onset of PE^44^. Notably, the methanogenesis pathway mediated by *Methanobrevibacter* has been shown to reduce serum TMAO levels, thereby reducing the risk of PE^45^. Considering the underlying mechanism, the results of human-based study and MR analysis, it is suggested that the GM genus *Methanobrevibacter* may play an important role in the development of HDP and could serve as a potential target for disease prevention and treatment.

Our study has some strengths. Firstly, this is the first study to explore the composition and longitudinal change of GM in HDP patients and healthy controls at three time points during gestation. Secondly, the nested case–control design can confirm the exposure (GM) is ahead of outcome (HDP). Thirdly, our finding was further validated by the comprehensive MR analysis which detected the causal relationship between *Methanobrevibacter* and HDP without some confounding factors and reverse causation.

Apparently, there are still several limitations. Firstly, the cohort included a total of 744 subjects, due to the low incidence rate of HDP and lost to follow-up bias, the sample size eligible for analysis was limited, thereby impacting the robustness and reliability of our findings. Secondly, our analyses were restricted to the genus level, rather than a more specific species level, owing to the limited resolution of 16S rRNA sequencing. Thirdly, this study did not compare the characteristic of gut microbiota between pregnant and non-pregnant women. Finally, this study could not clarify whether the genus *Methanobrevibacter* affects HDP through the BMI pathway or not. This issue could potentially be resolved through mediation analysis and additional RCTs involving human subjects.

In conclusion, by conducting a nested case–control study, we characterized the longitudinal alternation of GM in HDP patients during pregnancy and identified several differential microbial taxa. Followed by the MR analysis, we validated the protective causal effects of the *Methanobrevibacter* on PE risk. Our findings may promote the prevention and treatment of HDP as well as provide valuable insights into the pathogenesis of HDP from the perspective of GM. In the future, some biochemical indices and gut microbiota-derived metabolites could be analyzed to clarify the mechanism between the GM and HDP. And further randomized controlled trials by using probiotics like *Methanobrevibacter* could be conducted to confirm their protection effect on HDP.

## Methods

### Study population

From Mar 2017 to Dec 2018, the pregnant women who participated in the early pregnancy follow-up cohort in the Hunan Provincial Maternal and Child Health Hospital (no. ChiCTR1900020652) were enrolled in our nested case–control study. The inclusion criteria of the subjects were: (1) singleton, natural conception; (2) no history of diabetes, hypertension, thyroid disease, or cardio-cerebrovascular disease prior to pregnancy; (3) no recent acute infection, and no use of antibiotics during gestation. According to the 2013 American Obstetrics and Gynecology Standard^46^, GH was defined as hypertension found for the first time at 20 weeks in pregnancy. PE was defined as first elevated blood pressure with clinical proteinuria after 20 weeks in pregnancy. In addition to GH and PE, pregnancy complicated with chronic hypertension and chronic hypertension with preeclampsia should also be included in HDP, but because of the cohort inclusion criteria and limited sample size, only GH and PE were included.

In this cohort, 744 subjects were followed up during the early, middle, and late trimesters during pregnancy, completely. 46 HDP cases were diagnosed in the study cohort, including 29 GH cases and 17 PE cases. There were 19 and 3 cases of GH and PE with complete stool samples throughout the entire pregnancy period, respectively. To establish a control group, 30 pregnant women with normal blood pressure and complete stool samples were randomly selected from the original follow-up cohort. All participants provided informed written consent before taking part in the study. The study was approved by the Medical Ethics Committee of Hunan.

Provincial Maternal and Child Health Hospital (no. EC201624) and all methods were performed in accordance with the relevant guidelines and regulations.

### DNA Extraction and 16S rRNA Gene Sequencing

Using the Stool DNA Kit (Qiagen, Germany), total bacterial DNA was isolated from 160 to 180 mg of feces. The barcode-specific 515F-806R primer pairs were used to amplify the 16S rRNA gene V4 regions^47^. After the PCR reaction, products were combined in equal amounts and then purified with GeneJETTM Gel Extraction Kit (Thermo Scientific). The TruSeq DNA PCR-Free Library Preparation Kit (Illumina, USA) was used to create the sequencing libraries in accordance with the manufacturer's instructions. Finally, 250 bp paired-end reads were sequenced and produced through the Illumina HiSeq platform.

### 16S rRNA Gene Sequencing data analyses

The 16S rRNA gene sequence dataset was separated into individual files using QIIME2, then denoised and categorized into operational taxonomic units (OTUs) with DADA2 (at least 97% sequence similarity)^48^. The representative sequence for each OTU was checked on taxonomy annotation according to SILVA database^49^. Alpha diversity was measured by Shannon and Chao1 indices. Principal coordinates analysis (PCoA) and permutated analysis of variance (PERMANOVA) were utilized to cluster and visualize the samples as well as quantify the differences in beta diversity (representing the overall microbiome composition) between groups, respectively. Linear discriminant analysis effect size (LEfSe) was conducted to explore differential microbial features between groups^50^. Taxa with* P* < 0.05 and linear discriminant analysis (LDA) score > 2.0 were ultimately considered. Trend analysis for differential GM at three time-points during pregnancy was performed using the R package “profile”. Spearman's rank correlation was utilized to analyze the correlation between gut microbiota and blood pressure. All analyses were performed using R version 4.2.2 with the significant threshold setting as *P* < 0.05.

### MR analysis

#### Data source and instrumental variables selection

Publicly available GWAS summary meta-analysis statistics were obtained for GM and HDP (GH/PE). The GM GWAS dataset conducted by the MiBioGen consortium consisted of 24 multiple ancestry studies including 18,340 subjects^51^. Genetic association results for HDP were derived from the FinnGen research project, which involved more than 16 million SNPs from European ancestry participants. We extracted summary GWAS statistics for GH (4255 cases/114,735 controls) and PE (3556 cases/114,735 controls), respectively^52^. Detailed information on GM, GH, and PE GWAS datasets was summarized in Table S6.

**Figure 5:**
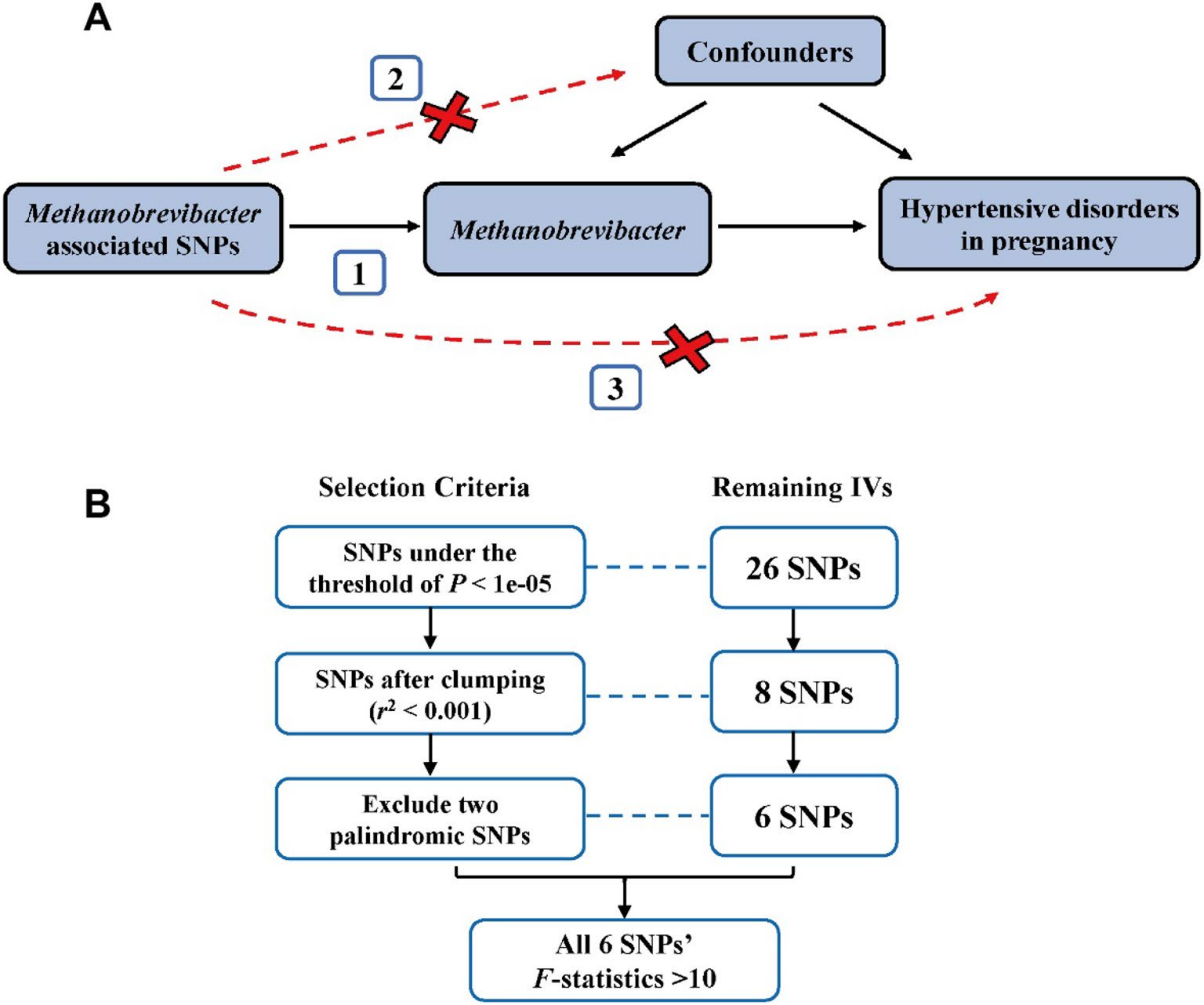
Schematic representation of the MR analysis. (**A**) The three assumptions of MR: (1) Relevance assumption: the genetic variants being used as instrument are associated with exposure (*Methanobrevibacter*). (2) Independence assumption: the genetic variants must not be associated with confounding factors. (3) Exclusion assumption: the genetic variants must influence outcome (HDP) only through the *Methanobrevibacter* pathway, not through other pathways. (**B**) The flowchart of instrumental variable selection. SNP, single nucleotide polymorphism; IV, instrumental variable.

To satisfy the three key assumptions of MR analysis (Fig. 5A), three steps were conducted to select the optimal instrumental variables (IVs): (1) SNPs under a locus-wide significance threshold of *P* < 1e-05 were obtained as potential IVs related to exposure trait^19^. (2) To ensure independence, we employed the PLINK clumping method with a window of 10,000 kb (*r*^2^ < 0.001) and excluded palindromic SNPs^53^. (3) SNPs with *F*-statistics < 10 were excluded to avoid the weak instrumental bias^54^ (Fig. 5B). Only the differential genera which fulfill the selection criteria could be used to perform the MR analysis.

### Statistical analyses

Using the R package “TwoSampleMR”, the inverse-variance weighted (IVW) method was set as the primary MR analysis to detect the causal associations between exposure (GM) and outcomes (GH and PE). The IVW method calculates the total causal effect by using the weighted linear regression model combined with the weight coefficient, under the condition that the intercept is zero^55^. Several other MR methods including Maximum Likelihood (MaxLik), Weighted Median (WM), and MR robust adjusted profile score (MR.RAPS) were also conducted to assess the robustness of our results^56–58^.

Cochran’s IVW Q statistics and leave-one-out analysis were used to identify potential heterogeneous IVs. MR-Egger intercept and MR Pleiotropy RESidual Sum and Outlier (MR-PRESSO) global test were conducted to test whether directional horizontal pleiotropy is driving the results of MR analyses^59,60^.

**Figure 6:**
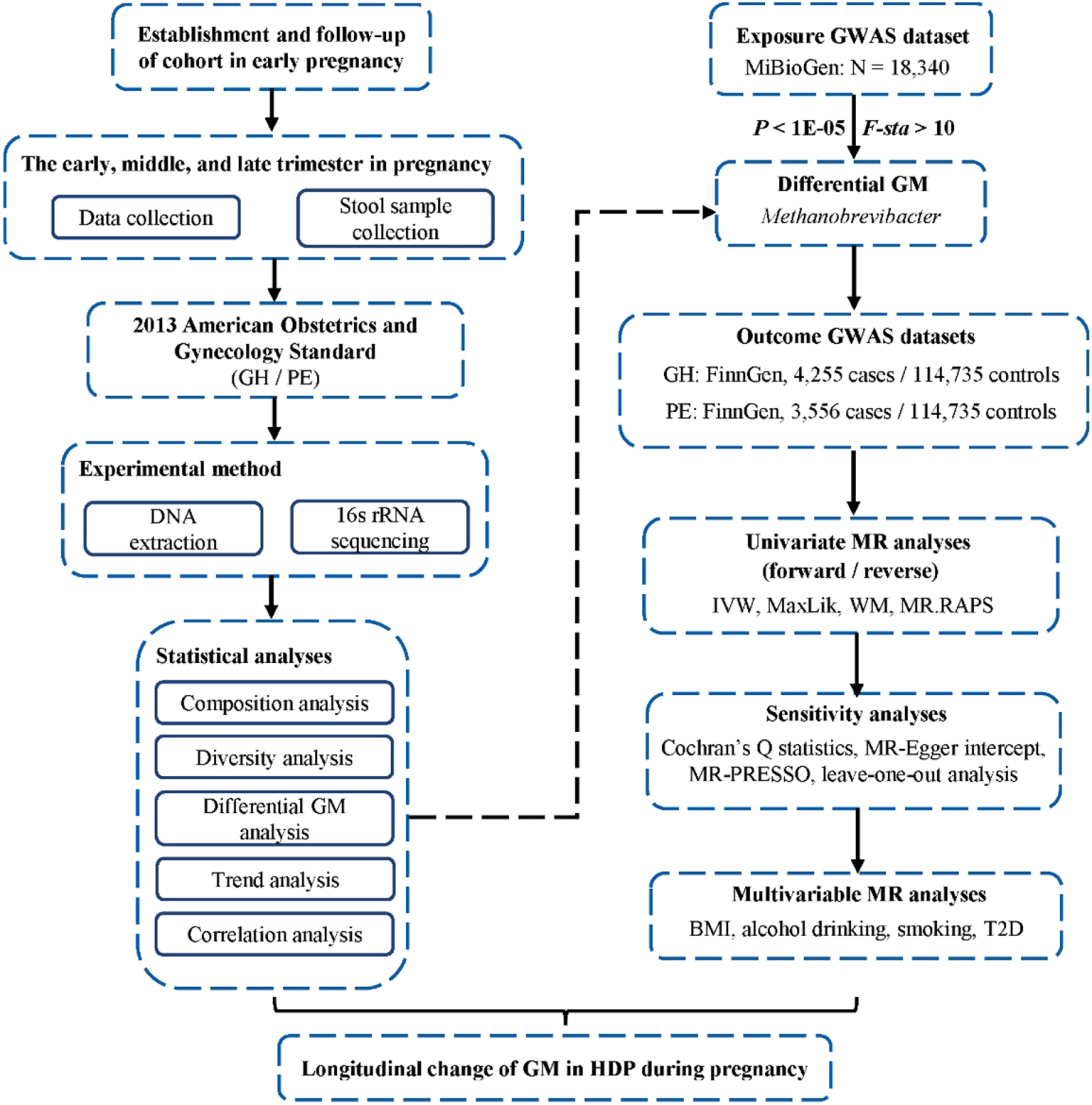
Flowchart of this study. HDP, hypertensive disorders in pregnancy; GH, gestational hypertension; PE, pre-eclampsia; GM, gut microbiota; GWAS, genome-wide association studies; MR, mendelian randomization; IVW, inverse-variance weighted; MaxLik, maximum likelihood; WM, weighted median; MR.RAPS, MR robust adjusted profile score; MR-PRESSO, MR Pleiotropy RESidual Sum and Outlier; BMI, body mass index; T2D, type 2 diabetes.

Reverse MR analysis was used to confirm the causal direction. The methods were similar to those of forward MR except for setting HDP as exposure and GM as outcome. Finally, we conducted multivariable MR (MVMR) analyses considering the possible confounders which may affect the outcome. The confounders including BMI (IEU number: ukb-b-19953), alcohol drinking (IEU number: ukb-b-5779), smoking (IEU number: ieu-b-4877), and T2D (IEU number: ebi-a-GCST006867). Flowchart of this study was shown in Fig. 6.

### Ethics declarations

The nested case–control study involving human subjects was approved by the Medical Ethics Committee of Hunan Provincial Maternal and Child Health Hospital (No.EC201624). Every subject provided written consent to participate in this study. No new approval and consent were required in MR analysis because those have been presented in the original GWAS.

### Supplementary Information

Supplementary Figures.

Supplementary Tables.

## Data Availability

All data generated or analyzed during this study are included in this published article (and its Supplementary Materials files).
