# Supplementary material for: Longitudinal change of gut microbiota in hypertensive disorders in pregnancy: a nested case–control and Mendelian randomization study
Source: Sci Rep. 2023 Oct 9;13:16986. doi: 10.1038/s41598-023-43780-w (PMC10562506; doi:10.1038/s41598-023-43780-w)
Supplement: Supplementary file 1 — Supplementary Figures. [file 41598_2023_43780_MOESM1_ESM.docx]

**Supplementary Figures 1-3**

**Longitudinal Change of Gut Microbiota in Hypertensive Disorders in Pregnancy: A Nested Case-Control and Mendelian Randomization Study**

Xinrui Wu^1,2^, Qi Li^3^, Jiawang Cai^1^, Houxiang Huang^1^, Shujuan Ma^4+^,

Hongzhuan Tan^2+^

Figure S1. Rarefaction curve.

Figure S2. Spearman rank correlation analysis between the differential GM and blood pressure during pregnancy.

Figure S3. Forest plot of MR analysis of the GM genus *Methanobrevibacter* on the risk of PE.


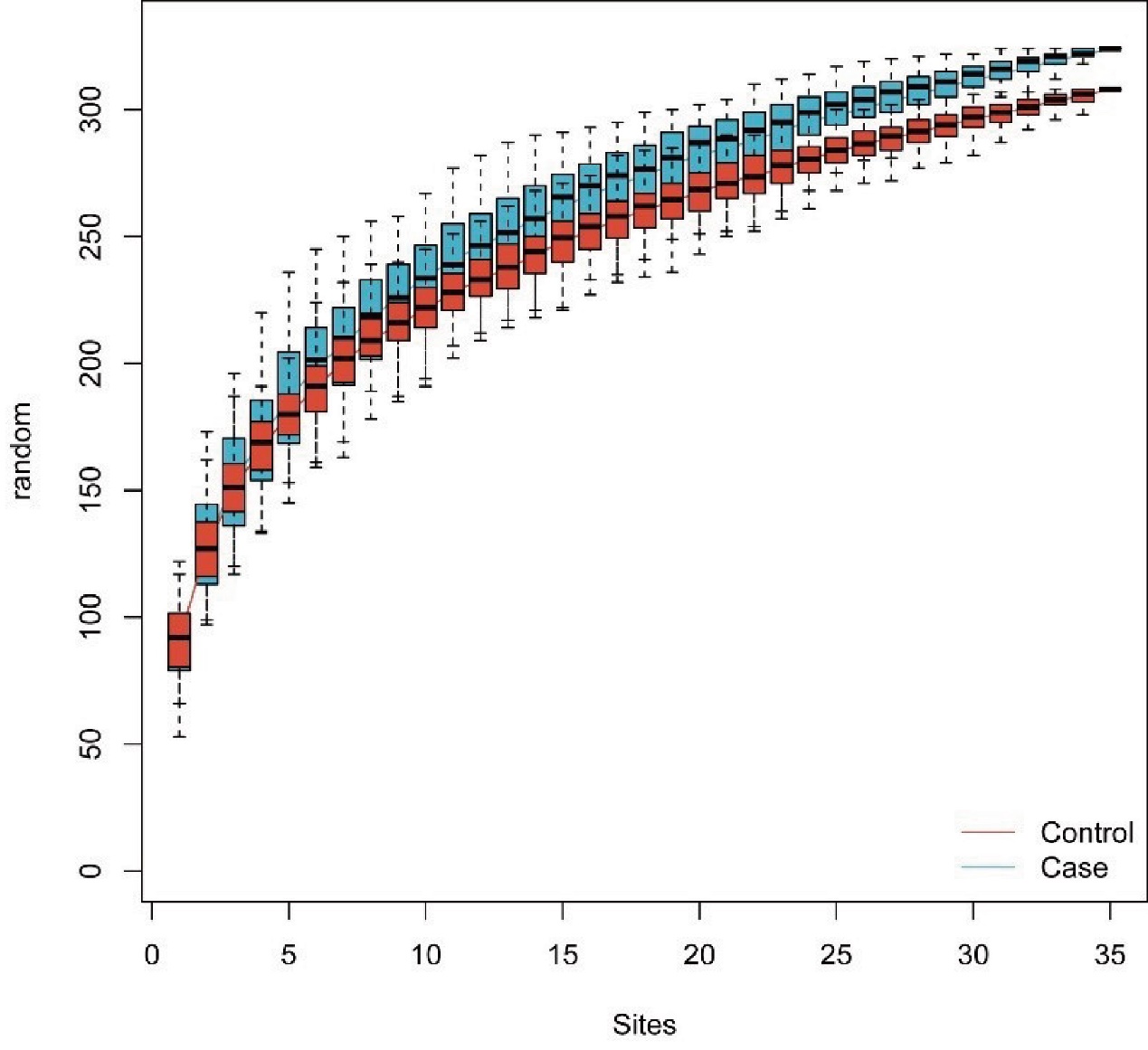


**Figure S1.** Rarefaction curve.


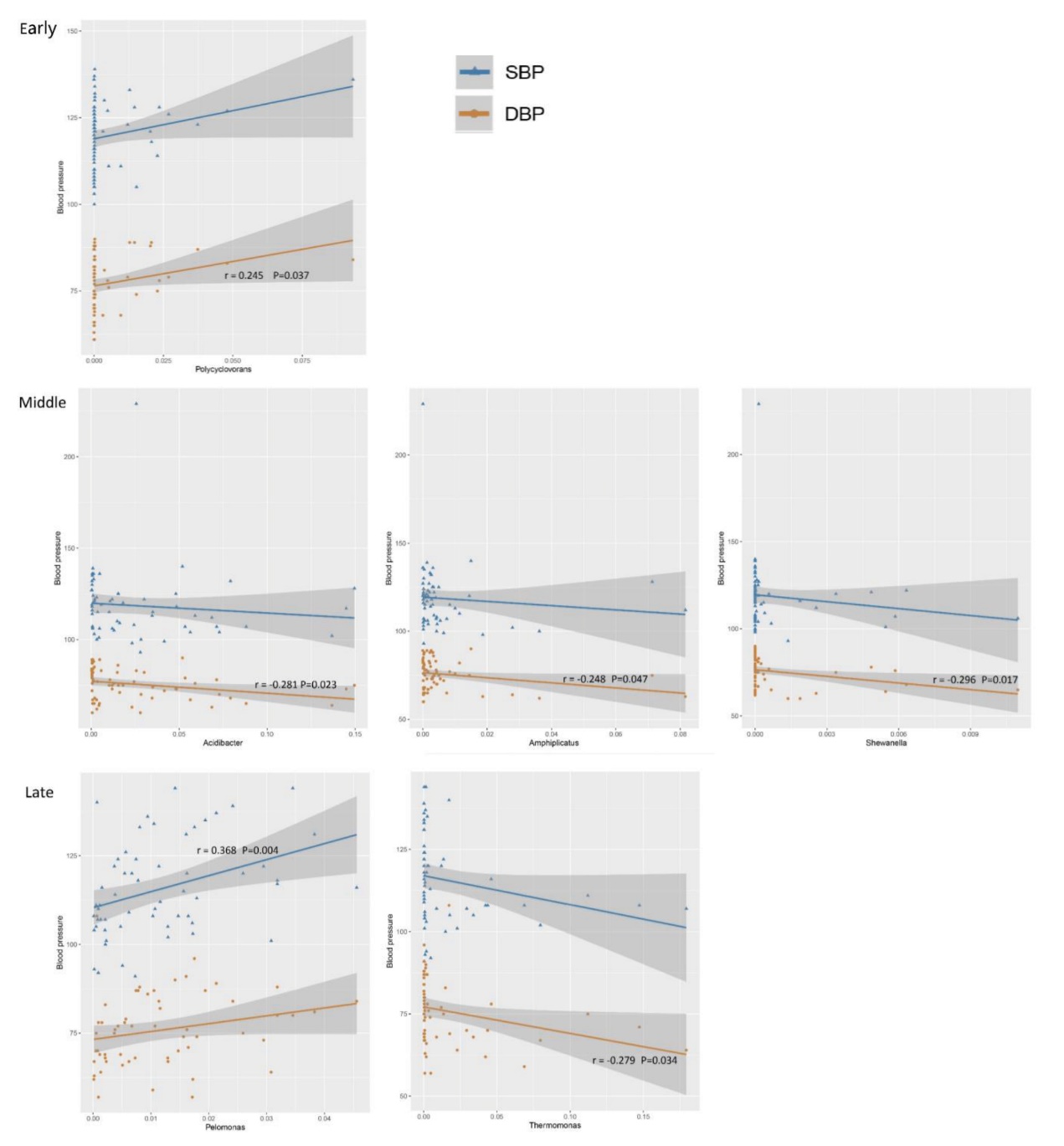


**Figure S2.** Spearman rank correlation analysis between the differential GM and blood pressure during pregnancy. SBP: systolic pressure; DBP: diastolic pressure.


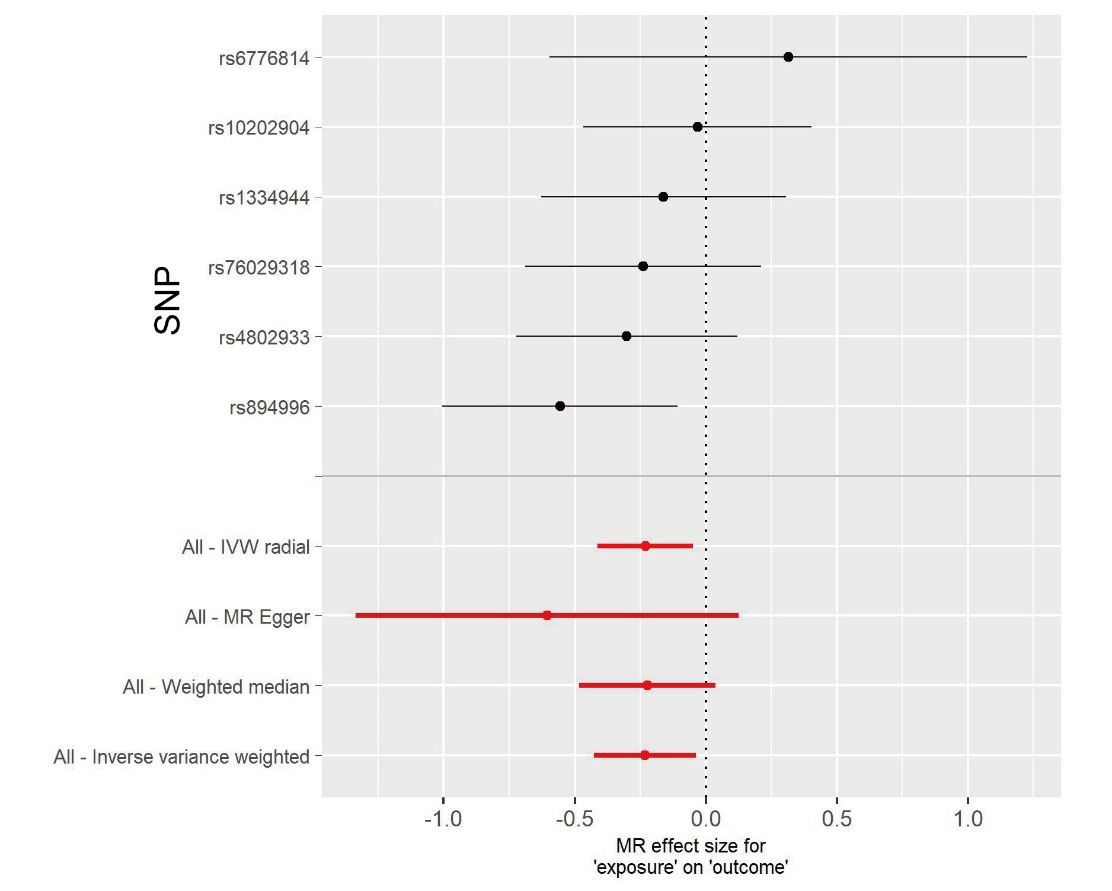


**Figure S3.** Forest plot of MR analysis of the GM genus *Methanobrevibacte*r on the risk of PE. The black points represent the estimated causal effect of individual SNPs, while the red points show the causal effect estimated using diverse methods that encompass all SNPs. Horizontal lines denote the 95% confidence interval. SNP, single nucleotide polymorphism.
